# The Potential and Cost of Carbon Dioxide Removal Using Direct Air Capture with Land-Based Wind and Utility-Scale Photovoltaics

**DOI:** 10.1021/acs.est.5c14628

**Published:** 2026-01-30

**Authors:** Elwin Hunter-Sellars, Tao Dai, Nathan C. Ellebracht, Hélène Pilorgé, Maxwell Pisciotta, Alexander P. Bump, Edna Rodriguez Calzado, Susan D. Hovorka, Corinne D. Scown, Simon H. Pang

**Affiliations:** † Materials Science Division, 4578 Lawrence Livermore National Laboratory, 7000 East Avenue, Livermore, California 94550, United States; ‡ Biological Systems & Engineering Division, Lawrence Berkeley National Laboratory, 1 Cyclotron Road, Berkeley, California 94720, United States; § Joint BioEnergy Institute, 5885 Hollis Street, Emeryville, California 94608, United States; ∥ Department of Chemical and Biomolecular Engineering, 6572 University of Pennsylvania, 220 South 33rd Street, Philadelphia, Pennsylvania 19104, United States; ⊥ Bureau of Economic Geology, 12330 The University of Texas at Austin, 10611 Exploration Way, Austin, Texas 78758, United States; # Energy Analysis & Environmental Impacts Division, 1666 Lawrence Berkeley National Laboratory, 1 Cyclotron Road, Berkeley, California 94720, United States; g Energy & Biosciences Institute, University of California, 282 Koshland Hall, Berkeley, Berkeley, California 94720, United States

**Keywords:** Carbon capture, wind and solar photovoltaic electricity, geologic storage, geospatial analysis, technology learning

## Abstract

The rapid deployment of direct air capture and storage (DACS) is critical for achieving emission targets, necessitating precise evaluation of the scale and cost of carbon dioxide removal. This study examines the availability of land, electricity generation, and geologic CO_2_ storage within the United States, estimating a technical potential for low-temperature, adsorbent-based DACS to remove approximately 9 gigatonnes of CO_2_ annually. By 2050, a substantial portion of this removal could be achieved at net-removed costs below $300/tonneCO_2_, though costs are highly variable depending on factors such as facility scale, construction expenses, climate-dependent productivity and heating efficiency, and geologic storage conditions. In the short term, DACS deployment will help identify key research priorities for advancing technology and reducing removal costs. Concurrently, there is an urgent need for scientifically robust and standardized frameworks for monitoring, reporting, and verifying DACS performance across both established and emerging technologies and energy sources.

## Graphical abstract

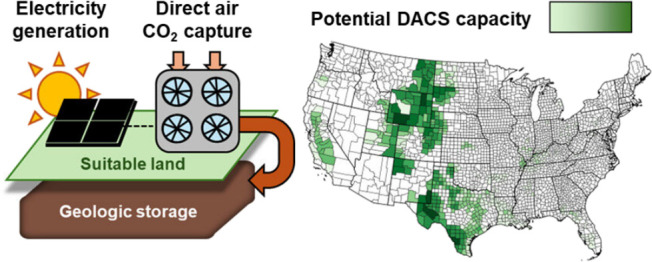

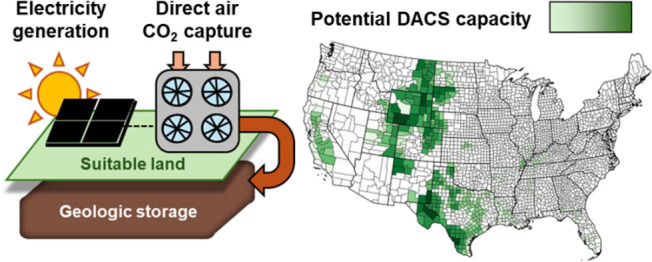

## 1 Introduction

Reaching global net emissions targets will require, beyond mitigation of existing CO_2_ emissions, rapid and expansive deployment of carbon dioxide removal (CDR) technologies to compensate for hard-to-abate emissions and address historic anthropogenic emissions. Recent analyses suggest the United States alone will require carbon removals at the gigatonne scale by 2050,^1,2^ likely achieved through a wide portfolio of CDR technologies.^3,4^ While nature-based CDR approaches such as afforestation and soil carbon sequestration have low costs, their scalability and permanence are limited.^2^ Consequently, engineered solutions—particularly direct air capture and storage (DACS)—are receiving attention, despite their higher costs, due to their potential for large-scale, permanent CO_2_ removal.^5,6^

The majority of DACS technologies deployed today can be categorized into solvent-based and adsorbent-based systems. Solvent-based DACS utilizes liquid solvents, typically aqueous hydroxides, that react with CO_2_ to form a carbonate, which is later converted to a solid and treated at temperatures approaching 900 °C to release high purity CO_2_ for storage.^7^ This regeneration is typically accomplished via oxycombustion of natural gas, and the air–liquid contact leads to substantial evaporative water losses, estimated at 1–9 tonnes water per tonne of captured CO_2_.^7^ In contrast, adsorbent-based DACS utilizes functionalized solids to capture CO_2_ in a two-step swing process, with regeneration occurring at lower temperatures (80–120 °C).^8^ This lower temperature requirement enables the use of a wider range of heat sources, while the use of a nonaqueous capture media reduces the process’ reliance on water feedstocks.^9,10^

Installing DACS at a meaningful scale will demand substantial investment in land, energy, natural resources, and capital. Approximately 80% of DACS’ energy consumption is dedicated to providing heat for regeneration,^11,12^ making access to low-cost, low-emission energy critical.^13^ Alongside electricity requirements, deployment of DACS is limited by the storage potential of geologic formations in the proximity of the capture facility. The United States possesses geologic storage potential at the teratonne scale,^14,15^ but this potential is highly spatially dependent^2^ and would require either colocation of DACS facilities with injection sites or the use of CO_2_ pipeline infrastructure.

Projections for DACS deployment scale vary significantly between studies. Fuhrman et al. determined that global DACS deployment in 2050 could vary from 0.01–12 gigatonnes per year, depending on the future scenario and priorities including societal and economic factors, land and energy usage, and international cooperation, among others.^16^ Fahr’s assessment of global DACS utilizing low-emission energy sources found that, based on global land and energy availability, DACS based on existing geothermal or bioenergy technologies could capture between 1 and 10 gigatonnes per year, while solar photovoltaics could facilitate hundreds of gigatonnes of DACS per year,^17^ i.e. far above many studies’ estimates of the required quantity of CDR.^1,18^ Adsorbent DACS facilities utilizing wind or solar photovoltaics would be operated entirely via electricity through the use of Joule heating,^19,20^ electric boiler-produced steam,^12^ or heat pumps.^8^ Assessments by Fauvel^21^ and Javadi^6^ estimate CDR deployment within the United States alone could reach between 1–2.3 gigatonnes per year by 2050, with DACS accounting for a significant portion of this removal. In both studies, DACS deployment was concentrated in central and southern states, particularly Texas due to its abundant geologic storage capacity, and would significantly impact local energy systems, requiring up to 50% of a state’s electricity and/or natural gas supply. A study by Edwards et al.^22^ suggests that gigatonne-scale deployment of DACS is possible if DACS has a similar technology growth rate to historical analogues like coal scrubbers and low-carbon electricity generation. While the deployment scale varies greatly depending on the historical analogue used, DACS deployment is concentrated in a few key regions, such as the United States, China, and Brazil, with the study acknowledging the influence of local policy on early and continued adoption of CDR.

The cost of DACS, similar to its deployment potential, is highly sensitive to the location. For example, a first-of-a-kind (FOAK) adsorbent-based DACS facility powered by intermittent wind and solar photovoltaics has been estimated to cost between $1091–9564 per tonne of CO_2_, decreasing to $97–1507 at the gigatonne scale, depending on the country of deployment.^23^ For FOAK facilities, regional variations in energy generation intermittency and the cost of materials and labor had the largest impact on cost, while technology learning and electricity prices were more impactful for facilities at the gigatonne scale. Similar regional cost variation has been observed in Europe, where DACS costs were estimated from €450–2,500 per tonne of CO_2_, largely driven by local energy prices, intermittency, and carbon intensity.^24^ Alongside regional factors, the DACS cost is strongly influenced by its deployment scale. In the early years of deployment, novel forms of CDR, such as DACS, will need to grow rapidly to reach relevant scales, as widespread deployment can drive down the cost of DACS through mechanisms such as market competition,^23^ public-private partnerships,^23^ and technology learning.^25^ The rate of deployment may be limited by cost, local policy, and funding incentives^26,27^ or the ability for a particular DACS technology to be scaled up.^28^ Technology learning rates, i.e. the ability to improve or drive down the cost of a technology as it is deployed, vary greatly between studies for adsorbent DACS,^2,23,25,29–31^ from 9.7–20% and 2.5–10% for capital and operating costs, respectively. This can result in a similarly wide range of future capture costs, between $96–386 per tonne of CO_2_.^32^

Given these uncertainties, accurately assessing DACS potential and cost requires a high-resolution, regionally explicit analysis that accounts for local constraints on land, energy, and storage. In this study, we investigate the near- and long-term deployment potential of adsorbent-based DACS (Ad-DACS) within the United States, focusing on systems powered by wind and solar photovoltaics. We employ high-resolution geospatial analysis to identify the intersection of: (1) available land, incorporating both physical (land type, slope) and social (protected status) constraints; (2) available electricity, considering purpose-built electricity generation and future technology expansion for the United States’ electrical grid; and (3) identified, quantifiable geologic storage. We quantify spatially explicit capture costs and deployment potentials at the county level, highlighting regions of opportunity for large-scale Ad-DACS deployment. We also assess the role of current grid electricity mixes in enabling and limiting DACS deployment. This work underscores the necessity of moving beyond “one-size-fits-all” solutions for carbon removal, emphasizing the importance of regional analysis in guiding effective and efficient DACS deployment strategies.

## 2 Methods

### 2.1 CO_2_ Capture Facility Process Model

A simplified Ad-DACS process model was developed using a modular vacuum-temperature-swing system based on an amine-based solid adsorbent coated onto square-channel monolithic contactors (Figure 1).^2,12^ Rather than dynamically representing transient adsorption and desorption processes, the model uses a fixed total cycle time and average CO_2_ removal efficiency (75%), as well as a sorbent mass-specific working CO_2_ capacity modulated by ambient climate conditions (temperature and humidity) and per-cycle oxidative degradation.^33^ A fixed thermal input for regeneration was used based on estimates from Climeworks,^34^ supplied via low-grade steam generated by an electricity-powered air-source heat pump. The outputs of the process model were sensitive to local ambient conditions due to their impact on CO_2_ capture productivity,^35^ heat pump performance, and fan energy. A summary of the relevant process parameters and associated references can be found in Table 1.

**Figure 1:**
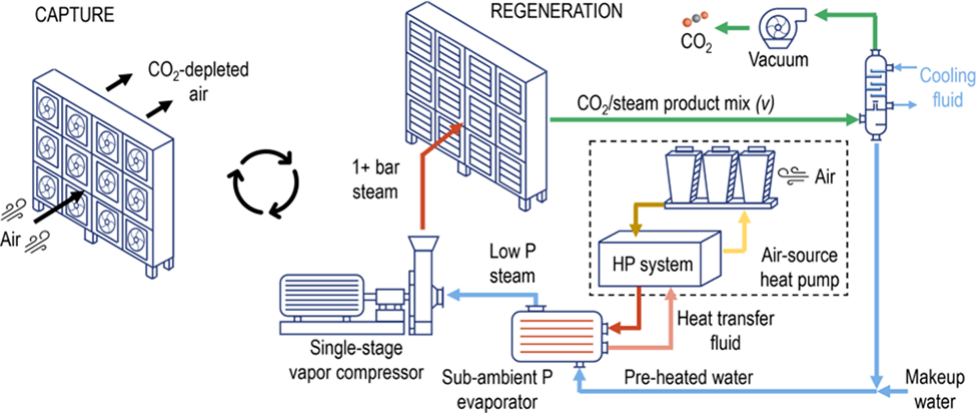
Schematic of mass and heat flow within the Adsorbent DACS process, wherein heat is provided via saturated steam, itself generated using an electric air-source heat pump,^2^ powered by grid or purpose-built wind or solar photovoltaic electricity.

**Table 1:** Summary of the Adsorbent DACS Process Parameters

Parameter	Units	Value
Baseline adsorbent cyclic working capacity (30 °C, 0% RH), *n*_*working,base*_	molCO_2_/kg_adsorbent_ cycle	0.8
Mass ratio of contactor to adsorbent	kg_contactor_/kg_adsorbent_	1.2
Cycle time	mins	20
Degradation rate constant, *k*_*degrad*_	%/cycle	5.1 × 10^–3^
CO_2_ capture capacity fade prior to sorbent replacement	%	30
Number of cycles at sorbent replacement, *cycles*_*rep*_		15363
Fan electricity requirement^12,36^	GJ/tonneCO_2_	1.1
Vacuum pump electricity requirement^12^	GJ/tonneCO_2_	0.6
Compressor electricity requirement^37^	GJ/tonneCO_2_	0.3
Regeneration steam requirement^34^	GJ/tonneCO_2_	11.9
Baseline capacity factor		0.9
Labor and maintenance	*% total OPEX*	4.5
Balance of plant CAPEX	*% total CAPEX*	10
Capital scaling (Lang) factor^12^	*$*_*overnight*_ */$*_*bare*_	4.5
Plant lifetime	*years*	20
Capital discount rate^12^	*%*	12.5

#### 2.1.1 Impact of the Local Environment on Adsorbent Productivity

To quantify the impact of temperature and humidity on adsorbent capture productivity, spatially explicit data at a resolution of 25 km^38,39^ was combined with interpolated estimates of productivity at different temperatures and dew points by Cai et al.^35^ The Cai et al. work uses model parameters for the adsorption and mass transfer of CO_2_ and water, determined experimentally under a range of conditions,^40,41^ to predict the binary CO_2_-water adsorption performance. For environmental conditions outside of the experimentally determined data set, performance was assumed to match the nearest valid condition. Productivity modulating factors (*k*_*climate,Cai*_ *(T,RH)*) were calculated by using local daily average conditions and then averaged over the operational days. The resulting location-specific annual productivity values (*n*_*working,avg*_) were combined with the baseline adsorbent working capacity to determine the mass of adsorbent required to meet the nameplate capacity of the facility. Capacity fade due to adsorbent degradation was accounted for via a per-cycle exponential decay factor;^42^ adsorbent was assumed to need replacement after losing 30% of its working capacity. To account for possible difficulties in operating a DACS facility under freezing conditions,^43^ the capacity factor of the DACS facility was reduced proportionately based on the number of days the local average daily temperature below was 0 °C. The functional working capacity was calculated by the following formula:

$$n_{working,avg}=n_{working,base}\times k_{c\lim ate,Cai}\left(T,RH\right)\times \frac{\overset{cycl{es}_{rep}}{\underset{cycle=0}{\sum }}{\left(1-k_{de\mathrm{grad}}\right)}^{cycle}}{cycles_{rep}}$$

#### 2.1.2 Energy for Regeneration

As adsorbent regeneration accounts for a large proportion of the DACS total energy requirement, the operating efficiency of the air-source heat pump will strongly influence the cost of the DACS facility. A correlation from Schlosser et al.^44^ was used to determine the heat pump coefficient of performance (COP), using a *T_out_* of 86 °C and a temperature lift determined by the local air temperature:

$$COP=a\times {\left(\Delta T_{lift}+2b\right)}^{c}+{\left(T_{out}+b\right)}^{d}$$

To stay within allowable temperature lifts for all ambient climate conditions, our process model used a heat pump system delivering heat to a subambient pressure (0.6 bar) water evaporator followed by a single-stage vapor compressor to compress the steam to saturation at 1.1 bar (102 °C).^45^ The total electrical input required for steam generation included the power required for the heat pump’s thermal load and the subsequent vapor compression. The full details of this thermal energy supply can be found in the Supporting Information.

#### 2.1.3 Impact of Elevation and CO_2_ Concentration on Fan Energy

To understand the impact of elevation, the local ambient pressure was calculated and used to determine the required quantity of air to be processed by the facility, which, in turn, altered the fan energy requirement. The same methodology was used for changes in the local ambient CO_2_ concentration.

### 2.2 Near-Term Deployment of Adsorbent DACS

Near-term Ad-DACS scenarios were assumed to be powered by grid electricity, using either US-average or state-level electricity prices and carbon intensities from 2023.^46^ Cost estimates were made considering a FOAK with a nameplate capacity of 100,000 tonneCO_2_/year on a gross-removed basis with adsorbent, heat pump, and fan performance modulated by local ambient conditions and capital costs modulated based on state-level construction cost coefficients.^47^ DACS facilities were assumed to be colocated with geologic storage, negating CO_2_ transport costs and assuming a geologic storage cost based on long-term projections.^2^ A summary of the state-level parameters can be found in Table S1.

### 2.3 Long-Term Deployment of Adsorbent DACS

For long-term deployment, a learning-by-doing analysis was carried out to project the capital cost and energy reductions in Ad-DACS processes to a target year of 2050, at which we assumed a global deployment of 0.5 gigatonneCO_2_/year to calculate the number of capacity doublings.^48,49^ The impacts of regional environmental conditions and construction cost coefficients on performance and cost were assessed similarly to those of the near-term scenario.

Technical potential in 2050 was constrained by suitable land availability,^50^ purpose-built wind and solar photovoltaic generation potential, and proximity to geologic storage (*vide infra*). In this configuration, the land footprint is assumed to be dominated by electricity generation, with the capture facility itself expected to account for a negligible quantity of the total land area.^51^

#### 2.3.1 Suitable Land Analysis

Land suitability criteria were focused on the requirements for land-based wind and utility-scale photovoltaics, chosen based on their projected low cost compared to other forms of low-emission energy.^52^ Both general and technology-specific siting criteria, summarized in Table 2, were applied onto the 30-m resolution 2019 National Land Cover Database (NLCD),^53^ serving as a reference layer of projection and processing steps for any geospatial analysis.

**Table 2:** Criteria Applied for Land Suitability of Renewable Electricity Generation for Adsorbent DACS

Condition	Notes
NLCD	Open water and wetlands excluded^53^
Protected land	Protected Areas Database, National Conservation Easement Database^54,55^
	3 km buffer distance for GAP^a^-status = 1, 2 (e.g., national parks)
	0 km buffer distance for GAP^a^-status = 3, 4 (e.g., state parks)
	3 km buffer distance for areas of critical environmental concern^56^
	3 km buffer distance for roadless areas^57^
Wetlands	0.3 km buffer distance^53^
Developed	0 km buffer distance^53^
Other developed	Airports: 3 km buffer distance^58^
	Railroads: 0.015 km buffer distance^58^
	Transmission lines: buffer distance based on voltage^58^
	Power plants: 3 km buffer distance^59^
	Buildings: 0.3 km buffer distance^60^
	Wind turbines: 3 km buffer distance^61^
Wind and solar photovoltaic expansion	Excluded land overlapping with prioritized wind and solar photovoltaic electricity generation development area^62^
Slope (Solar)	Slope <5% (2.86°)^63^
Slope (Wind)	Slope <20% (11.31°)^63^
Co-location (Solar)	Excluded lands with forests, pasture/hay, or cultivated crops occupying >25% of ∼5 km^2^ grid space^53^
Co-location (Wind)	Excluded lands with forests occupying >25% of ∼5 km^2^ grid space^53^
Contiguity	<5 km^2^ contiguous area excluded

a GAP = Gap Analysis Project.

General criteria were grouped into four categories: land identified as ‘protected’ by the government; open water and wetlands; developed lands, including buffer zones around man-made installations; and land that is projected to be occupied by low-emission electricity generation for electrical grid expansion as identified by Denholm et al. “All-Options” scenario.^62^ The resulting land class data were resampled into a 2250-m resolution grid point map (Figure S1) to improve computational efficiency.

Technology-specific criteria were then applied, based on the highest generation potential for each grid point. The first criterion excluded land with slopes above 5 and 20% for wind and solar, respectively. The second criterion excluded land based on their colocation with certain NLCD land classes. Wind development was excluded in spaces containing more than 25% forest, and solar development was excluded in areas with more than 25% of forest, pasture/hay, or cultivated crops.^53^ In Alaska, solar photovoltaics was excluded as an electricity generation option because of low solar resources in that region.^64^ Finally, a minimum contiguous land area of 5 km^2^ was specified to remove isolated pixels and indirectly constrain a minimum land area for power generation, DACS, and storage infrastructure and facilities. For additional details of this land suitability analysis, refer to Dai et al.^50^

#### 2.3.2 Wind and Solar Photovoltaic Electricity Potential and Cost Analysis

At each of the resampled grid points *i*, the electricity generation potential *E*_*i*, *j*_ in MWh per year with technology *j* (*j* is wind or solar) can be calculated as

$$E_{i,j}=CF\times 8760\times A_{i,j}\times {PD}_{j}$$

where *CF* is the temporal capacity factor, *A*_*i,j*_ is the quantity of suitable land, in km^2^, and *PD_j_* is the power density of the technology. In this study we assumed a constant power density for each technology based on literature values: solar photovoltaics was assumed to have a power density of 45 MW/km^2^, a conservative estimate based on the median value from Bolinger and Bolinger;^65^ wind was assumed to have a power density of 4.3 MW/km^2^ based on the mean value in a multiyear analysis by Harrison-Atlas et al. which focused on the United States and considered the potential advancements in turbine manufacturing technologies.^66^ Levelized costs of energy (LCOE) were calculated using the Electricity Annual Technology Baseline’s 2050 Moderate scenario.^52^ Spatially explicit estimates of LCOE were based on resource class (Tables S2, S3), i.e. wind speed at a 120-m height and global horizontal irradiance for wind and solar electricity respectively,^67,68^ the cost of utility-scale energy storage for each technology, and the regional construction cost coefficient. An energy storage duration of 8 and 10 h was chosen for wind and solar technologies, respectively.

#### 2.3.3 Geologic Storage Potential and Cost

For this analysis, Ad-DACS facilities were assumed to be constructed only on land with geologic storage potential. While CO_2_ transport has been considered using several methods,^69^ we elected to exclude it from our scope due to its projected cost and uncertainty. Spatially explicit geologic storage capacities and cost were taken from Pett-Ridge et al.,^2^ which took into account factors related to injectivity, CO_2_ plume, and pressure area and the costs associated with project exploration. Regions with poorly defined storage potential or cost and electricity generation grid points overlapping these regions were excluded from further analysis.

## 3 Results and Discussion

### 3.1 Near-Term Cost of Adsorbent DACS

We estimated state-specific costs for near-term, FOAK (100,000 tonneCO_2_/year scale) Ad-DACS facilities (Figure 2). We found that an Ad-DACS facility utilizing grid electricity could capture and store CO_2_ at a cost as low as $840/tonneCO_2_, when accounting for the CO_2_ emissions of the electricity supply. The largest contributors to cost in the near-term are the capital costs of supplying and replacing adsorbent modules and the capital and electricity costs of the heat pump used to supply steam for adsorbent regeneration.

**Figure 2:**
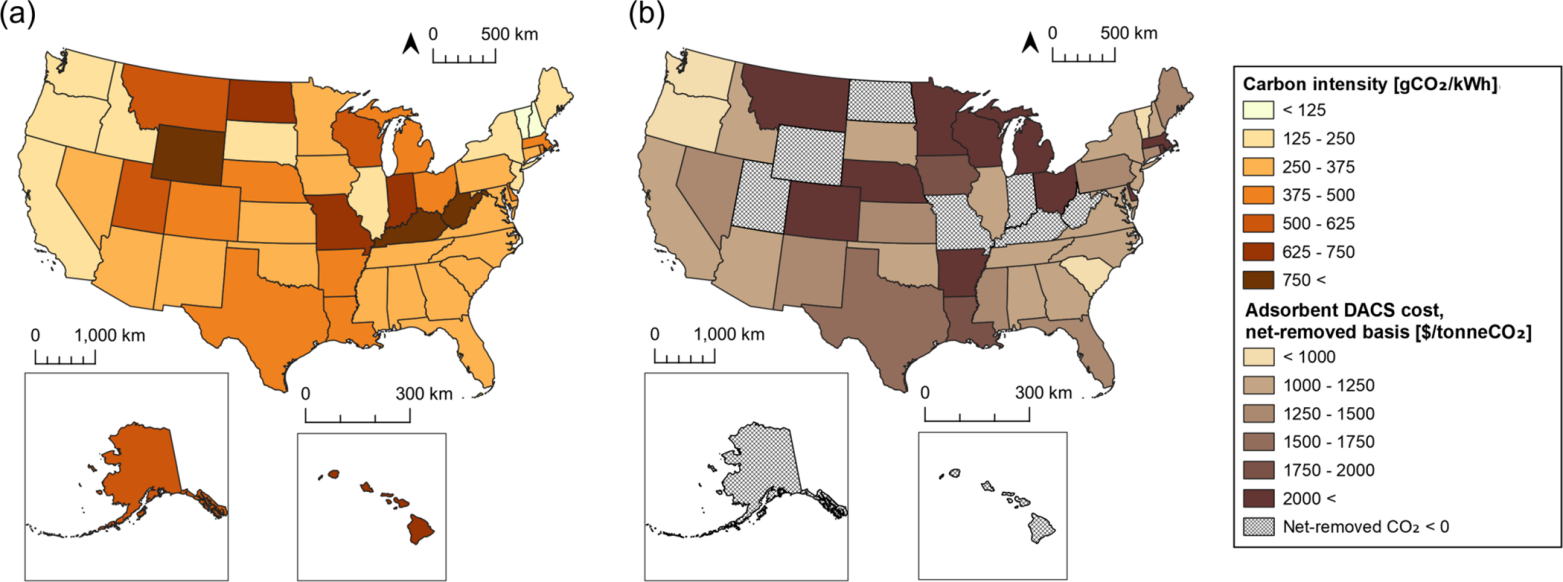
(a) 2023 state-level electrical grid carbon intensities; (b) near term, state-level costs for Adsorbent DACS facilities on a net-removed basis, powered by the state’s grid electricity. Costs were impacted by state-level grid carbon intensities, building cost coefficients, and local climatic conditions. Calculations assume a FOAK facility size of 100,000 tonneCO_2_/year.

The cost of DACS on a net-removed basis is strongly impacted by the state in which it is deployed due to variations in temperature and relative humidity, state-level construction costs, and the carbon intensity and electricity price of the electrical grid. When accounting for the net-removed CO_2_ based on the energy demand and carbon intensity of the energy supply, the average cost of near-term Ad-DACS is around $2850/tonneCO_2_, based on the US-average grid carbon intensity and electricity price. This estimate sits well within the cost ranges estimated by previous studies and is heavily impacted by the net removal fraction of 0.24 resulting from the energy-associated CO_2_ emissions.^23,70^

#### 3.1.1 Impact of Local Grid Electricity Price and Carbon Intensity

The local cost of electricity purchased from the grid strongly influences the operating costs of the facility due to the high energy demand of Ad-DACS. The total energy costs, the majority of which are associated with steam generation via an air-source heat pump system, account for between 27–52% of the total cost of capture in the continental United States. The proportion jumps to 71% in Alaska due to the poor efficiency of an air-source heat pump in a cold climate.

The emissions associated with energy generation strongly impact the quantity of net-removed CO_2_, i.e. the quantity of CO_2_ captured by DACS minus the quantity of CO_2_ released via energy generation, and the subsequent cost of the DACS facility on a net-removed basis. The carbon intensity of the electrical grid varies strongly by state due to differences in the energy source and power plant efficiency. For example, based on 2023 numbers,^71^ Vermont’s hydroelectric-powered grid generates only 3.6 g of CO_2_ released per kWh of electricity, which results in a net-removed cost of $840/tonneCO_2_. On the other hand, Michigan’s heavily natural gas-powered grid generates 414 g of CO_2_ per kWh of electricity, resulting in a net-removed cost of $5100/tonneCO_2_. In several states, such as Wyoming and Alaska, CO_2_ emissions from electricity generation exceed the quantity of CO_2_ captured using that electricity for DACS, resulting in overall positive emissions and an undefined net-removed cost. Similar results were demonstrated by Sendi et al.^72^ who found that net CO_2_ removal could not be achieved in India, China, South Africa, and the central United States due to their grid carbon intensities. Postweiler et al. discussed the potential of operating DACS flexibly, i.e. prioritizing operation when the carbon intensity of the electrical grid is low.^73^ While this study cited cost and efficiency benefits, this level of process optimization is outside the scope of our work. Regardless, these results further demonstrate the need to carefully consider the CO_2_ emissions of a region’s existing electrical grid if DACS is planned to utilize it and illustrates the importance of grid technology mixes.

#### 3.1.2 Impact of Local Environmental Conditions on Performance

Temperature and humidity impact both the productivity of the sorbent and the coefficient of performance of the air-source heat pump (Figure S2). Increasing temperature led to a reduction in productivity, as the exothermic nature of CO_2_ adsorption is thermodynamically favored at lower temperatures.^40,74^ At a fixed temperature, productivity was found to increase with relative humidity up to a certain point, typically between 40 and 50%, before plateauing or decreasing with increasing humidity. While the presence of water can increase the amine efficiency of the adsorbent,^75^ longer regeneration times are required to completely desorb both CO_2_ and water, reducing productivity.^35^ Based on these relationships, DACS facilities deployed in colder states with mild levels of humidity, such as Montana and Idaho, possessed the highest capture productivity, resulting in lower adsorbent capital costs due to the reduced quantity of adsorbent required to meet a nameplate capacity of 100,000 tonneCO_2_/year. Increasing the temperature reduced the required temperature lift of the electric heat pump, increasing its coefficient of performance and reducing the quantity of electrical energy required to meet the thermal energy requirements of the adsorbent regeneration. This led to heat pump electricity requirements between 4.1 and 6.6 GJ/tonneCO_2_ for the warmest (Hawaii) and coldest (Alaska) states respectively which, alongside the local electricity price, can have a strong impact on operating costs. The local ambient pressure and CO_2_ concentration impact the quantity of air that must be processed by the facility to meet a nameplate CO_2_ removal capacity, which in turn impacts the energy requirement of the facility’s fans. This is most noticeable in regions far above sea level, such as Colorado, Wyoming and Utah, where the low ambient pressure can increase near-term costs by up to 13%. Similar effects are observed with the CO_2_ concentration, although the regional variance is much smaller than for ambient pressure.

#### 3.1.3 Impact of Construction Cost Coefficients

The location in which a facility is constructed will impact the cost of that construction, based on factors such as local costs of labor and materials, labor availability, logistics, weather, and seismic and climatic conditions.^47,76^ The majority of the continental United States has cost coefficients between 0.8 and 1.2, which can result in up to 50% variation in capital costs for facilities constructed in different states (Table S1). Hawaii and Alaska, with coefficients of 2.2 and 2.7, respectively, have significantly higher cost coefficients due to their relative lack of interconnected infrastructure, labor and materials, and their unique climate and geography. On average, an Ad-DACS facility constructed in Alaska would cost approximately 113% more than one in the continental United States, without accounting for capture productivity or grid carbon intensity.

### 3.2 Long-Term Deployment of Adsorbent DACS with Purpose-Built Electricity Generation

Future Ad-DACS deployment was estimated based on the quantity of wind and/or solar photovoltaic electricity generation that could be constructed intersecting geologic storage. The cost of this Ad-DACS was based on the cost of electricity generation and the capital and operating costs of the facility itself, impacted both by technology learning and by regional economic and environmental conditions.

#### 3.2.1 Identifying Suitable Land for Electricity Generation

As shown in Figure 3(a), the criteria that resulted in the largest above-ground land exclusions were regions containing or adjacent to wetlands (1.74 million km^2^), regions with excessive slope (0.53/1.69 million km^2^ for wind/solar), and land prioritized for grid expansion and energy security (1.07/0.67 million km^2^ for wind/solar). Exclusion type was strongly related to the NLCD land classification within a region. For example, forests and shrublands made up approximately 38 and 35% of protected lands but only 27 and 7% of lands on or around wetlands, respectively.

**Figure 3:**
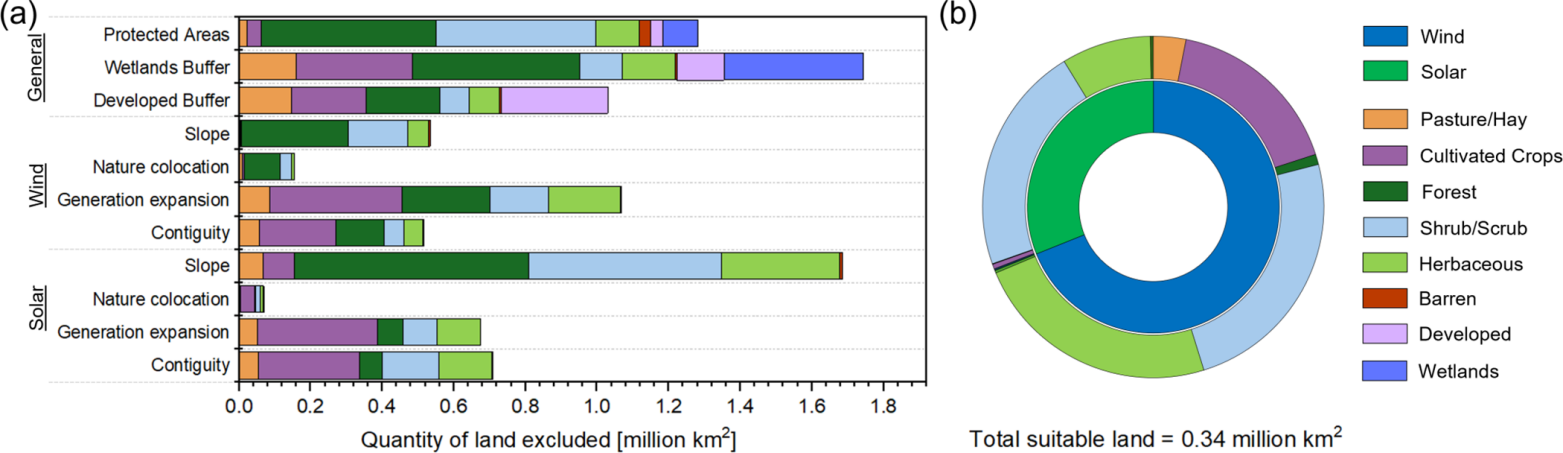
(a) Quantity of land excluded by general and technology-specific above-ground siting criteria; (b) land-class distribution of area intersecting electricity generation and geologic storage.

It is important to note that future demands for electricity generation, such as data centers and other industrial electrification,^77,78^ could require significant expansion of wind and solar photovoltaic electricity generation. While these factors were not accounted for in detail due to the difficulty to predict the quantity and location of the low-emission technologies required for buildout of these facilities, the ‘Generation expansion’ land exclusions prevents this analysis from double-counting land marked for electricity generation expansion by Denholm et al.^62^

After applying above-ground general and technology-specific siting criteria (Figure S3), approximately 0.75 and 0.45 million km^2^ was identified as suitable for wind and solar photovoltaic electricity generation respectively (Figure 4). States surrounding and east of the Rocky Mountains such as Montana and Wyoming have high potential for wind, as does north and southwest Alaska, while southwestern states such as Texas, New Mexico, and Arizona have high potential for solar. These analyses were conducted at a 30-m resolution, higher than other studies analyzing siting of wind^79,80^ and solar^80^ electricity generation, with resolutions between 90–100 m, which can lead to differences in the determined quantity of suitable land (Figure S4), and results in a more conservative estimate of the quantity of suitable land for electricity generation.

**Figure 4:**
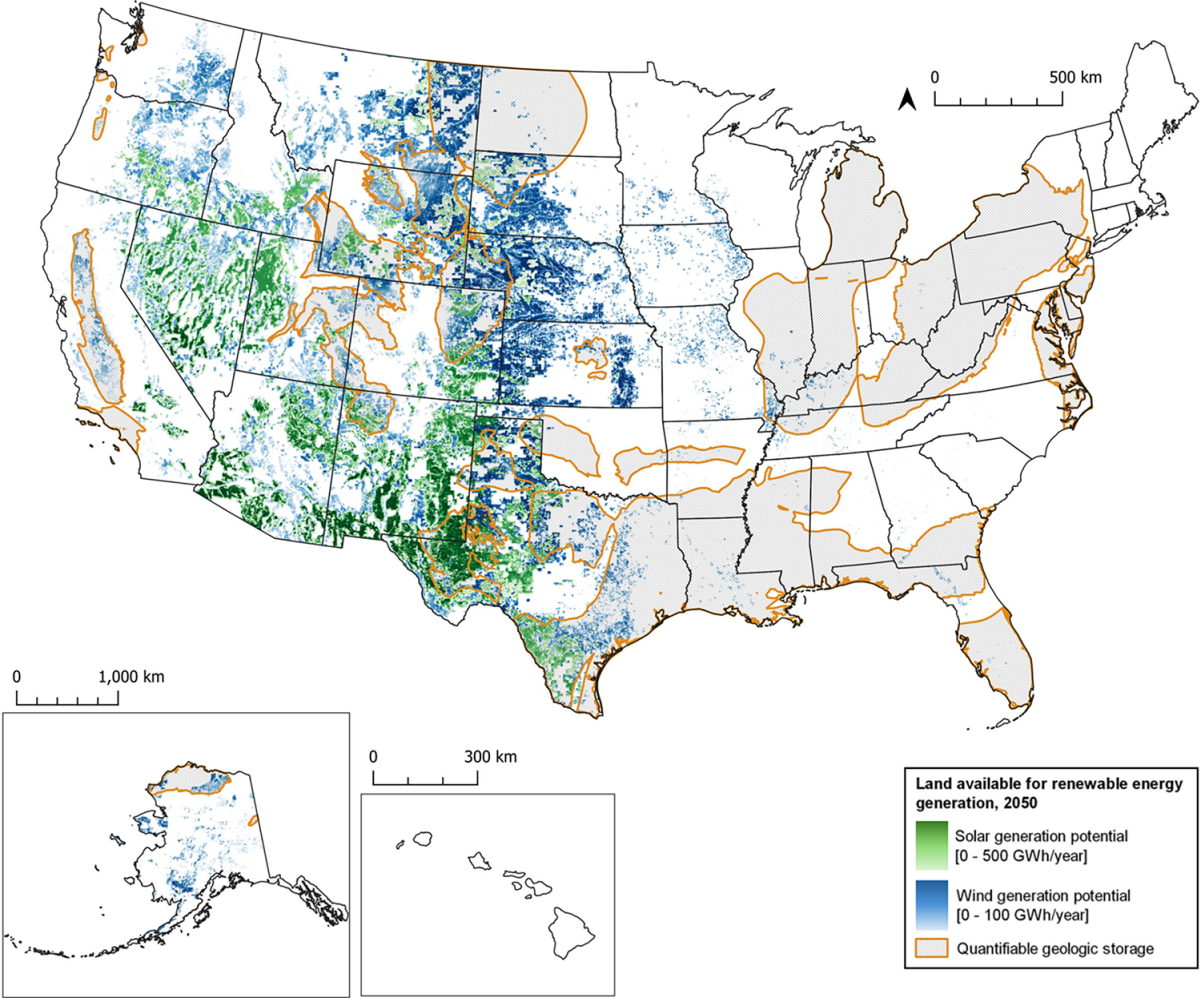
Identified suitable land for wind (blue) and solar photovoltaic (green) electricity generation for Adsorbent DACS facilities in the long term deployment scenario and the overlap with quantifiable geologic storage. Installations are color graded based on their generation potential, with darker colors indicating higher potential.

#### 3.2.2 Intersection with Geologic Storage

When considering only generation above geologic storage with quantifiable cost and capacity, approximately 0.34 million km^2^ could be utilized for electricity generation for Ad-DACS (Figure 3(b)). This land accounts for 16% of the total land area in the contiguous United States and would enable the generation of 41 and 10 PWh by solar and wind installations, respectively. In regions where both wind and solar energy could be produced, the technology with the higher generation potential was prioritized, although selecting based on cost produced similar results (Figure S5). Much of the intersection between electricity generation and storage is of the ‘Herbaceous’ or ‘Shrub/Scrub’ land class and is located in south and west Texas, Wyoming, and Colorado, with storage costs per tonne of CO_2_ varying between $4–40, with an average of $8. The requirement for colocation with geologic storage eliminated several regions within the western United States with significant potential for electricity generation, such as northern Nevada and southern Arizona—both of which, due to their low population density, have relatively little land prioritized for meeting the future load requirements of the electrical grid.^62^ These regions demonstrate the benefits of expansive mass and energy transport networks for future grid needs, energy security, and direct air capture.

#### 3.2.3 Long-Term Capture Potential for Adsorbent DACS

Based on the quantity of electricity generation colocated with storage, we estimate that, in the long term, approximately 2.5 and 6.8 gigatonnes of CO_2_ per year could theoretically be captured utilizing Ad-DACS powered by purpose-built wind and solar photovoltaic electricity generation, respectively, including utility-scale energy storage. This value accounts for seasonal variations in CO_2_ capture productivity and learning-based improvements to the energy efficiency of the capture and regeneration process. While most regions in the United States have some potential to accommodate Ad-DACS facilities, the largest opportunities for deployment are concentrated in North Slope, Alaska; Sweetwater, Campbell, and Carbon counties, Wyoming; Lea and San Juan counties, New Mexico; and Pecos, Reeves, Webb, and Culberson counties, Texas. Texas alone could accommodate over 2 gigatonnes per year of Ad-DACS capacity, in agreement with previous studies,^6,21^ largely due to its land area and intersection with geologic storage. It is important to note that this study reports technical potentials, i.e. if all available and appropriate lands were utilized for electricity generation purpose-built for DACS; we do not aim to imply that each region would reasonably deploy this much DACS. Identifying high priority regions for deployment can be helpful due to the catalytic impact of initial deployments on future adoption and cost of DACS.^22^

#### 3.2.4 Long-Term Regional Variations in Adsorbent DACS Cost

For long-term cost estimations, a blend of component-specific learning rates was considered for the pieces of equipment comprising Ad-DACS^31,34,81–83^ and applied to a learning curve up to a deployed capacity scale of 0.5 gigatonneCO_2_/year in 2050. Components with high degrees of modularity, or that are based on emerging science and technology such as the contactor and adsorbent media, were generally assigned higher learning rates,^23,82^ which are reflected in the reductions in cost between near- and long-term (Figure 5). Sievert et al.^32^ used a similar methodology for an Ad-DACS process, assigning learning rates ranging from 3–27% based on the novelty of the component.^32^ Using moderate rates for all components (Table 3) resulted in an overall learning rate of 9.7%, relatively conservative compared to other studies.^23,32,84^ While the adsorbent and contactor were assigned the highest learning rate based on its novelty compared to components such as vacuum pumps and compressors, it was assumed not to benefit from economies of scale due to its modularity. In addition to learning rates on capital expenditures, we applied learning to the thermal energy requirement, which was set to a target value of 7.025 GJ/tonneCO_2_ at a projected deployed capacity of 0.5 gigatonneCO_2_/year. This estimate is based on conservatively achieving 75% of the thermal energy requirement reduction targets of Climeworks, reported by Deutz and Bardow.^34^ This thermal requirement is higher than several short- and long-term estimates^12,31^ but lower than experimentally determined requirements at lab-scale, as would be expected. The average total post-learning electricity requirement for Ad-DACS was determined to be approximately 5.2 GJ/tonneCO_2_, similar to publicly reported estimates of energy requirements for current DAC plants by Carbon Engineering and Climeworks^25^ and on the more conservative end of estimates for long-term capture facilities.^12,23,31^

**Table 3:** Parameters for Technology Learning of the Adsorbent DACS Process

Component	Full range	Moderate
Adsorbent and contactor	10–15%	12%
Heat pump	5–12%	10%
Fans	2.5–7.5%	5%
Vacuum pumps	0–2.5%	0%
Dryers and compressors	0–2.5%	0%
Thermal energy requirement	5.4–8.65 GJ/tonneCO_2_	7.025 GJ/tonneCO_2_

**Figure 5:**
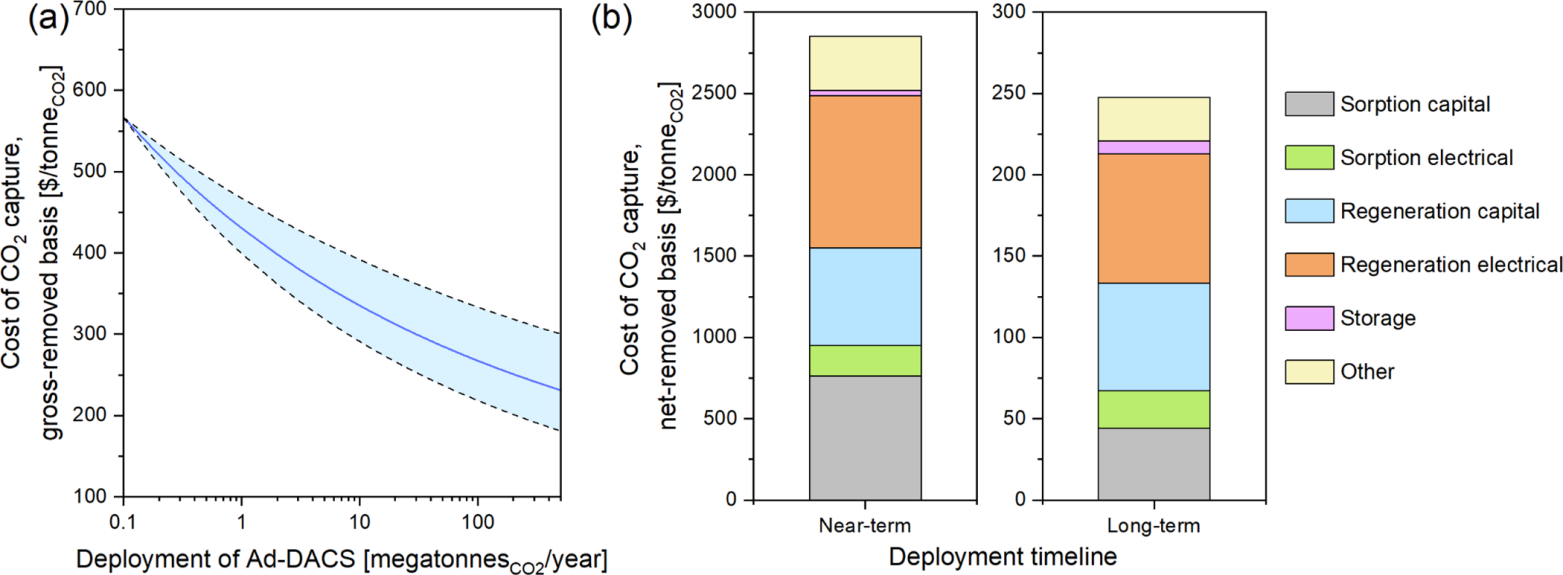
(a) Learning curves for Adsorbent DACS for deployment up to 0.5 gigatonneCO_2_/year on a gross-removed basis; dark blue line indicates a ‘moderate’ learning rate. Learning curves were calculated using baseline long-term values for electricity pricing and an energy carbon intensity based on the average between wind and solar photovoltaics. (b) Net-removed cost breakdown of near-term, grid-powered Adsorbent DACS, using US-average electricity carbon intensity and price, and post-moderate-learning Adsorbent DACS powered by wind or solar photovoltaic electricity. Both cases assumed plant scales of 100,000 tonneCO_2_/year, identical climate conditions, and a construction cost coefficient of 1.0. The long-term case assumed an energy carbon intensity based on the average between wind and solar photovoltaics.

After applying moderate learning, Ad-DACS’s net-removed cost was reduced from $2850/tonneCO_2_ in the near-term to $250/tonneCO_2_ in the long term. This cost reduction reflects not only the reductions in capital and operating costs from improvements to the technology and process (Figure 5(a)) but also transitioning from utilizing US-average grid electricity to low carbon intensity wind and solar photovoltaics, improving the net removed fraction of the Ad-DACS from 0.24 to 0.97.

When accounting for regional variations, capture costs vary from $210–980/tonneCO_2_ within the contiguous United States, while costs in Alaska varied from $1560–1730/tonneCO_2_, reflective of higher construction costs and lower heat pump COP (Figure 6). The lowest costs were concentrated in the southern United States, with the 25 lowest cost counties being in western Oklahoma and northern, western, and central Texas. Texas counties possess some of the highest technical potential capacities: Reeves and Pecos counties alone could theoretically support 0.23 gigatonneCO_2_/year at an average cost of $230/tonneCO_2_. The long-term Ad-DACS cost was influenced by several locational factors: electricity price, based on the local resource class and energy generation technology; construction cost coefficients; local ambient pressure and CO_2_ concentration and their influence on fan energy requirements; the temperature- and humidity-dependent adsorbent productivity and heat pump efficiency; and the cost of CO_2_ storage. The long-term cost of Ad-DACS in this study was more sensitive to the learning rate than regional factors, although total cost variations did not exceed ±6% in a 10% sensitivity analysis (Figure S6). Costs were also sensitive to the assumed global deployment scale, which has been hypothesized to vary based on allowable temperature rises, sociopolitical and industrial priorities, and technology mixes.^6,16,21^ Varying global deployment from 1–10,000 megatonneCO_2_/year reveals average Ad-DACS cost between $430–196/tonneCO_2_ on a net-removed basis (Figure S7). This cost reduction compared to the near-term estimates is largely due to the utilization of wind or solar photovoltaics, which have significantly lower carbon intensity compared to the current United States’ electrical grid. However, these differences in cost based on deployment scale incentivize prioritizing facility construction in regions with advantageous conditions, not only to reduce that facility’s cost but also to accelerate deployment scale and learning-induced cost reductions for future facilities in other, less-advantageous regions. Due to the significant electricity demand, the states with the lowest cost for electricity and geologic storage are generally expected to have the lowest DACS costs, particularly Texas, Oklahoma, and Wyoming. Several of these states were considered relatively undesirable in the near term for Ad-DACS deployment based on Figure 2, due to their carbon intensive electrical grid. However, the long-term analysis emphasizes the importance of low-emission electricity and its ability to significantly expand the potential of Ad-DACS within the United States.

**Figure 6:**
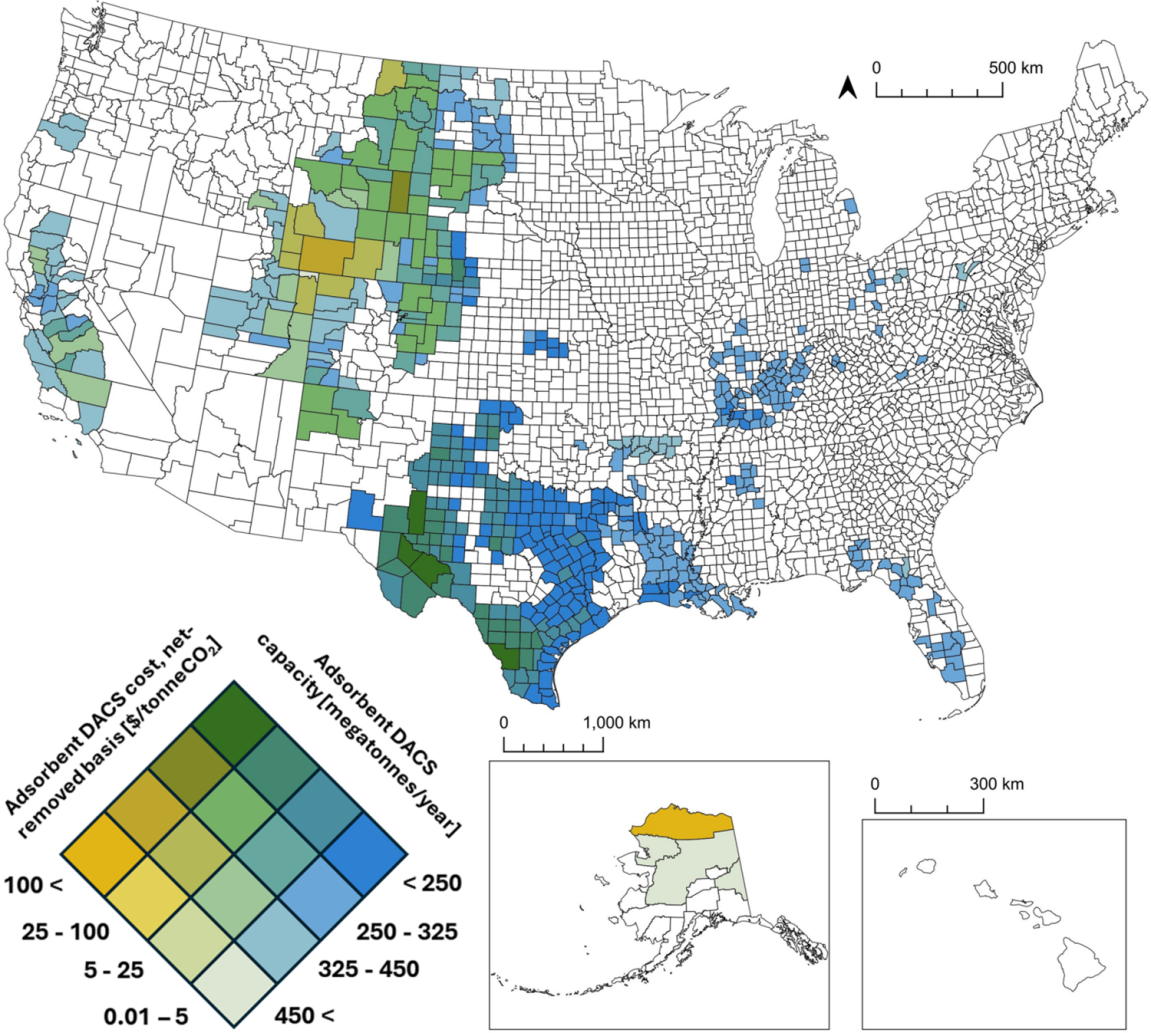
County-level assessment of potential long-term cost and capacity of Adsorbent DACS powered by wind or solar photovoltaic electricity, colocated with geologic storage.

### 3.3 Outlook for Adsorbent DACS Deployment in the United States

These analyses provide a foundation for further study on direct air capture and storage across the globe, not only within the United States. Challenges with heat supply and the current carbon intensity of the United States’ electrical grid further support the need for expanding low-emission electricity generation for the electrical grid, not only to reduce emissions but also to improve the efficiency of capture processes utilizing electrified processes, as well as providing energy security. In the near-term, the viability of grid electricity for powering DACS is strongly dependent on location due to the energy mix and carbon intensity of each state’s electrical grid. States like Vermont and Washington have relatively low capture costs in the near-term due to their high proportion of low-emission generation technologies, resulting in net-removed capture efficiencies of 99 and 81% respectively, but geologic storage availability makes these locations challenging in the absence of robust and inexpensive CO_2_ transportation networks or other CO_2_ storage mechanisms, e.g. via mineralization in basalts. On the other hand, states with ample geologic storage resources such as Texas and Wyoming have much lower capture efficiencies due to their emission-intensive electrical grids.

After applying moderate component learning rates to the DACS process, we estimate that in the long term, the continental United States could support 9.3 g of CO_2_ removal per year via Ad-DACS, utilizing purpose-built wind and solar photovoltaic electricity generation. Much of this capacity is concentrated in several states (Texas, Wyoming, New Mexico, California, and Alaska) due to a combination of land availability and suitable conditions for electricity generation. Long-term capture costs in the range of $210–1730/tonneCO_2_ were calculated, with regions such as Texas, Oklahoma and Wyoming having substantially lower costs due to their low electricity and storage costs. The high-resolution geospatial analysis emphasizes the need for region-specific research, as local environment and terrain are likely to have a strong impact on both the performance of a capture facility and the cost for constructing and operating one. However, this work identifies a number of high potential regions for long-term DACS deployment at a gigatonne scale, which could cement its role in achieving net emissions targets.

## Supplementary Material



## Notes

Information related to the process model, cost and capacity calculations, and methodology for Adsorbent DACS deployment in the short- and long-term, including supporting figures (PDF)

## ABBREVIATIONS

CDR: carbon dioxide removal
CO_2_: carbon dioxide
COP: coefficient of performance
DAC: direct air capture
DACS: direct air capture and storage
FOAK: first-of-a-kind
GAP: Gap Analysis Project
NLCD: National Land Cover Database
