# Supplementary material for: The Potential and Cost of Carbon Dioxide Removal Using Direct Air Capture with Land-Based Wind and Utility-Scale Photovoltaics
Source: Environ Sci Technol. 2026 Jan 30;60(7):5430–41. doi: 10.1021/acs.est.5c14628 (PMC12947619; doi:10.1021/acs.est.5c14628)
Supplement: Supplementary file 1 [file es5c14628_si_001.pdf]

Supplementary Information

The potential and cost of carbon dioxide removal using direct air capture with land-based wind and utility-scale photovoltaics

Elwin Hunter-Sellars,[a,‡] Tao Dai,[b,c,‡] Nathan C. Ellebracht,[a,‡] Hélène Pilorgé,[d] Maxwell Pisciotta,[d] Alexander P. Bump,[e] Edna Rodriguez Calzado,[e] Susan D. Hovorka,[e] Corinne D. Scown,[b,c,f,g,] Simon H. Pang [a,]***

[a] Materials Science Division, Lawrence Livermore National Laboratory, 7000 East Avenue, Livermore, CA 94550, USA

[b] Biological Systems & Engineering Division, Lawrence Berkeley National Laboratory, 1 Cyclotron Road, Berkeley, CA 94720, USA

[c] Joint BioEnergy Institute, 5885 Hollis Street, Emeryville, CA 94608, USA

[d] Department of Chemical and Biomolecular Engineering, University of Pennsylvania, 220 South 33rd Street, Philadelphia, PA 19104, USA

[e] Bureau of Economic Geology, The University of Texas at Austin, 10611 Exploration Way, Austin, TX 78758, USA

[f] Energy Analysis & Environmental Impacts Division, Lawrence Berkeley National Laboratory, 1 Cyclotron Road, Berkeley, CA 94720, USA

[g] Energy & Biosciences Institute, University of California, 282 Koshland Hall, Berkeley, Berkeley, CA 94720, USA

‡ these authors contributed equally to this work

Email: * cdscown@lbl.gov; ** pang6@llnl.gov

1. Steam generation for adsorbent regeneration

2. State-level costing parameters

3. Grid re-sampling

4. Resource classes of electricity generation

5. Map data related to near-term, state-level Ad-DACS costs

6. Land exclusions and data resolution

7. Generation technology prioritization

8. Cost sensitivity of near- and long-term Ad-DACS

9. Limitations of this study

10. References

1. Steam generation for adsorbent regeneration

As described in the main text, heat for adsorbent regeneration was supplied via low-grade saturated steam at 1.1 bar. To reduce the electrical requirement of generating this steam, our process model used an air-source heat pump featuring a sub-ambient pressure (0.6 bar) steam evaporator and a single-stage vapor compressor to compress the generated steam from 0.6 to 1.1 bar (102 °C). The total thermal regeneration energy (11.9 GJ per tonne CO_2 in near-term) was assumed to correspond to the total steam enthalpy at supply pressure (1.1 bar).

$$steam\ required(t_{steam}/t_{CO2}) = \frac{regeneration\ heat\ (GJ\ steam/t_{CO2})}{total\ steam\ enthalpy\ (GJ/t_{steam})}$$

The electricity required to produce this steam is broken down into the heat pump electrical work (W_{HP}) and the vapor compression work (W_{compr}). The heat pump thermal output, Q_{HP}, was determined by the latent heat of vaporization of water at the sub-ambient evaporation pressure (0.6 bar). The heat pump thermal output was used to size the heat pump system and to determine the electrical work required for the heat pump based on the location-specific COP.[1]

$$Q_{HP} = latent\ heat\ of\ evaporation\ (GJ/t_{steam}) * steam\ required\ (t_{steam}/t_{CO2})$$

$$W_{HP} = \frac{Q_{HP}}{COP}$$

The specific compression energy, W_{compr}, was calculated using the shaft work based on the specific volume, v, and pressure, p, of the steam [2]:

$$W_{compr,steam}\ (GJ/t_{steam}) = \int_{p_1}^{p_2} v dp$$

$$W_{compr}\ (GJ/t_{CO2}) = W_{compr,steam}(GJ/t_{steam}) * steam\ required\ (t_{steam}/t_{CO2})$$

$$W_{total} = W_{HP} + W_{compr}$$

2. <u>State-level costing parameters</u>

Table S1. United States state-specific electricity prices, electrical grid carbon intensities, and construction cost coefficients.

State	2025 electricity purchase price [US$/MWh][a]	2023 grid carbon intensity [$kgCO_2/MWh$][b]	Construction cost coefficient [c]
Alabama	7.21	330	0.835
Alaska	19.20	535	2.718
Arizona	7.74	310	0.935
Arkansas	7.49	448	0.910
California	16.89	200	1.205
Colorado	9.13	478	1.037
Connecticut	10.97	261	1.136
Delaware	8.66	497	1.121
D.C.	8.97	425	1.04
Florida	8.72	360	0.858
Georgia	7.40	332	0.859
Hawaii	30.90	655	2.175
Idaho	7.28	168	0.988
Illinois	8.32	237	1.078
Indiana	8.42	679	0.916
Iowa	7.56	352	0.988
Kansas	8.41	346	0.911
Kentucky	6.78	790	0.914
Louisiana	7.08	438	0.887
Maine	10.88	194	1.077
Maryland	9.64	253	0.995
Massachusetts	17.30	419	1.180
Michigan	8.76	414	1.021
Minnesota	9.45	363	1.102
Mississippi	6.78	371	0.798
Missouri	8.10	687	0.949
Montana	7.11	511	1.084
Nebraska	8.27	500	0.913
Nevada	6.86	297	1.150
New Hampshire	15.74	122	1.080
New Jersey	12.19	235	1.192
New Mexico	7.02	344	0.933
New York	7.22	237	1.127

State	2025 electricity purchase price [US$/MWh][a]	2023 grid carbon intensity [$kgCO_2$/MWh][b]	Construction cost coefficient [c]
North Carolina	7.00	290	0.879
North Dakota	8.40	641	1.118
Ohio	7.46	474	0.961
Oklahoma	6.27	294	0.909
Oregon	6.80	156	1.168
Pennsylvania	7.45	297	1.090
Rhode Island	18.30	384	1.138
South Carolina	6.92	253	0.926
South Dakota	9.14	167	0.982
Tennessee	6.28	324	0.846
Texas	6.97	388	0.894
Utah	7.05	614	1.093
Vermont	12.97	4	1.001
Virginia	7.40	269	0.973
Washington	6.62	132	1.142
West Virginia	6.92	873	0.972
Wisconsin	8.69	521	1.092
Wyoming	7.78	845	1.022

[a] U.S. Energy Information Administration, EIA-861, 2021, Total Electric Industry, Industrial price,[3] projected to 2025 from 2021 (14% increase).

[b] U.S. Energy Information Administration, 2023, State Electricity Profiles, Table 1: Summary Statistics.[4]

[c] US DoD Facilities Pricing Guide (UFC 3-701-01).[5] Average of area cost factors by state (Table 4-1, CONUS).

3. Grid re-sampling

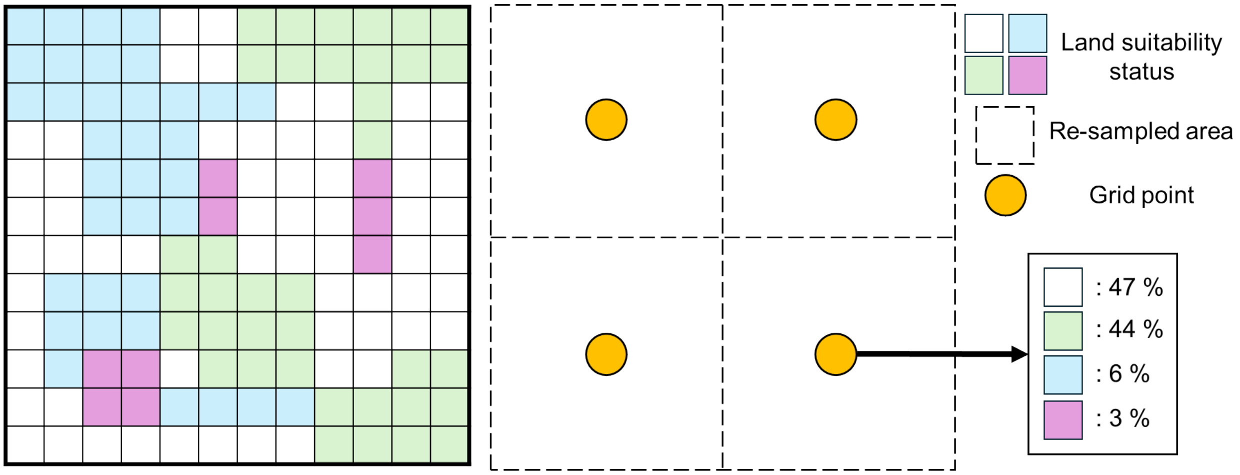

Figure S1. Schematic depicting the process of aggregating higher resolution dataset into lower resolution dataset with corresponding grid points. This resampling process retains the information of the fraction of suitable land contained within each re-sampled area within each grid point.

4. Resource classes of electricity generation

Table S2. Land-based wind resource classes and levelized cost of electricity.[6]

Type	Min. wind speed [m/s]	Max. wind speed [m/s]	LCOE [$/MWh]
Class 1	9.01	12.89	15.4
Class 2	8.77	9.01	16.6
Class 3	8.57	8.77	17.0
Class 4	8.35	8.57	17.5
Class 5	8.07	8.35	18.0
Class 6	7.62	8.07	18.9
Class 7	7.10	7.62	20.8
Class 8	6.53	7.10	23.3
Class 9	5.90	6.53	27.9
Class 10	1.72	5.90	42.5

Table S3. Utility-scale solar photovoltaic resource classes and levelized cost of electricity.[6]

Type	Min. GHI [a] [kWh/m^2/day]	Max. GHI [kWh/m^2/day]	LCOE [$/MWh]
Class 1	5.75	-	13.2
Class 2	5.50	5.75	13.6
Class 3	5.25	5.50	14.3
Class 4	5.00	5.25	15.1
Class 5	4.75	5.00	16.1
Class 6	4.50	4.75	16.8
Class 7	4.25	4.5	17.6
Class 8	4.00	4.25	18.5
Class 9	3.75	4.00	19.4
Class 10	0	3.75	21.2

[a] Global horizontal irradiance

5. Map data related to near-term, state-level Ad-DACS costs

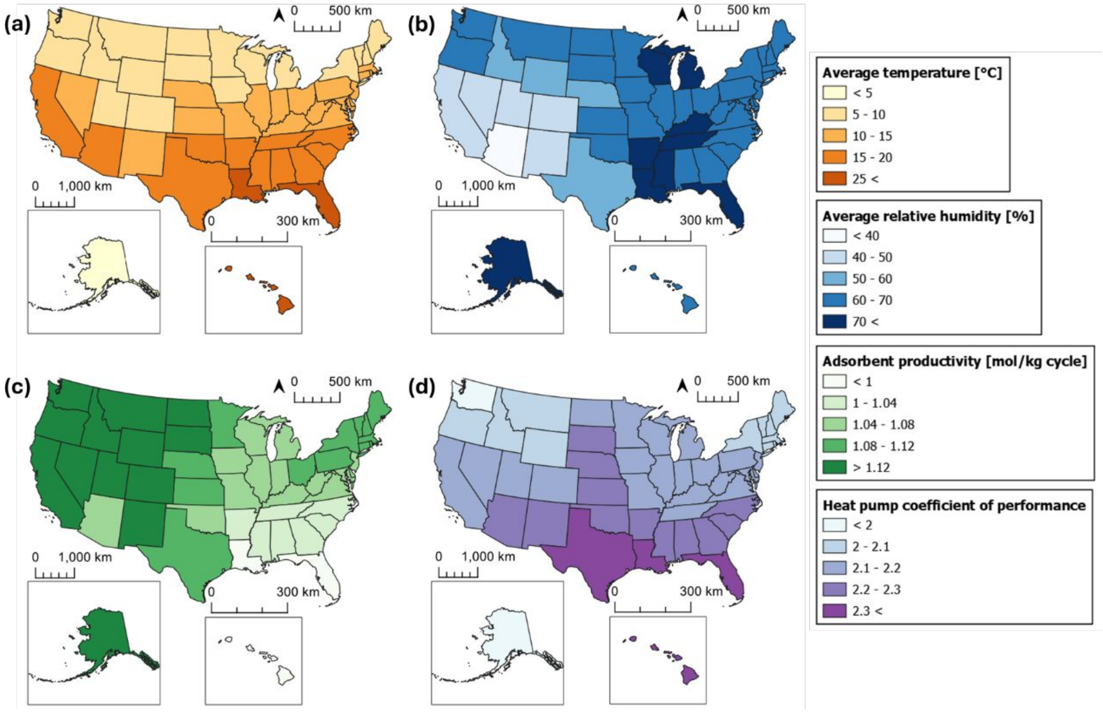

Figure S2. State-level average (a) temperature, (b) relative humidity, (c) adsorbent productivity and (d) electric heat pump coefficient of performance.[7] Productivity values were varied based on the impacts of temperature and relative humidity put forward by Cai *et al* [8]. Heat pump coefficients of performance were based on the correlation from Schlosser *et al.*, a function of the temperature lift and output temperature [1].

6. Land exclusions and data resolution

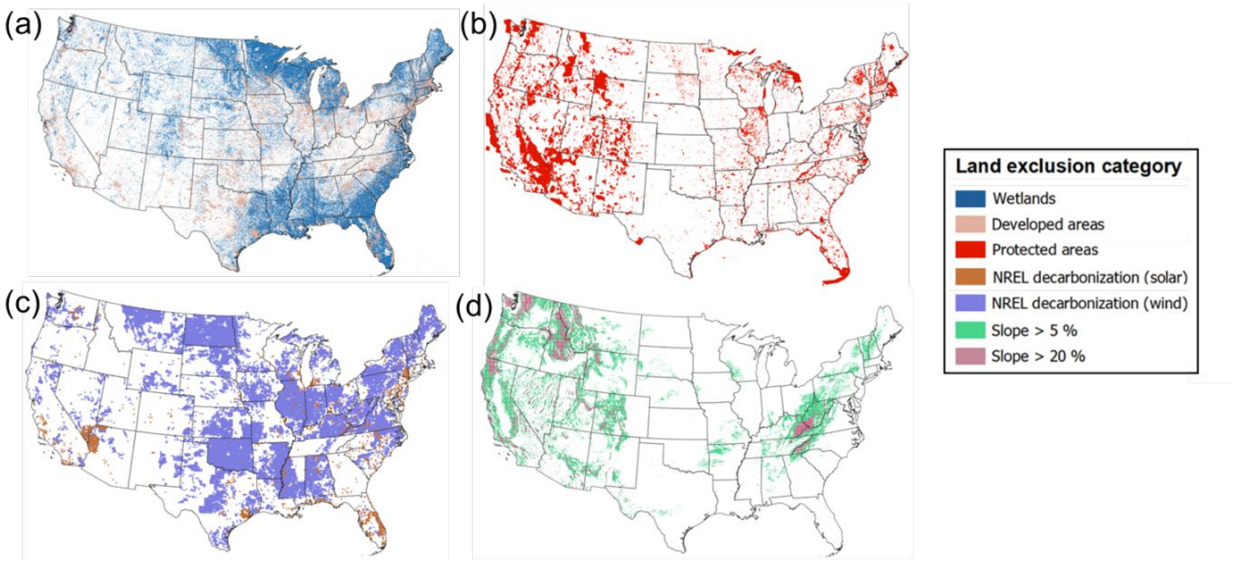

Figure S3. Land exclusions applied to electricity generation deployment for adsorbent DACS in the continental United States: (a) wetlands and developed area buffers; (b) protected areas; (c) lands prioritized for grid technology expansion, based on analyses by the National Renewable Energy Laboratory;[9] (d) land slopes exceeding allowable values for wind and solar deployment.

The resolution of the geospatial analysis can have several impacts on the determined quantity of suitable land. First, pixels of 'unsuitable' land use classes that would be excluded from use, due to merging with neighboring pixels, remain in the dataset. As a result, more pixels/land are identified as not suitable due to the land cover type. This results in a higher level of fragmentation in the land and more potential discontinuity, reducing the quantity of contiguous land. Additionally, the slope calculated can vary based on the resolution. Buakhao and Kangrang found that, for the same area, the slope calculated at 90-meter resolution is 25% percent lower than the slope calculated at 30-meter resolution [10]. Finally, roads, railways, and low-voltage transmission lines disappear at lower resolutions and are therefore not properly accounted for. The retention of smaller land class features at higher resolutions leads to more exclusions based on the 5 km^2 contiguous land condition compared to lower resolution. These factors result in a more conservative estimate of the quantity of suitable land for electricity generation.

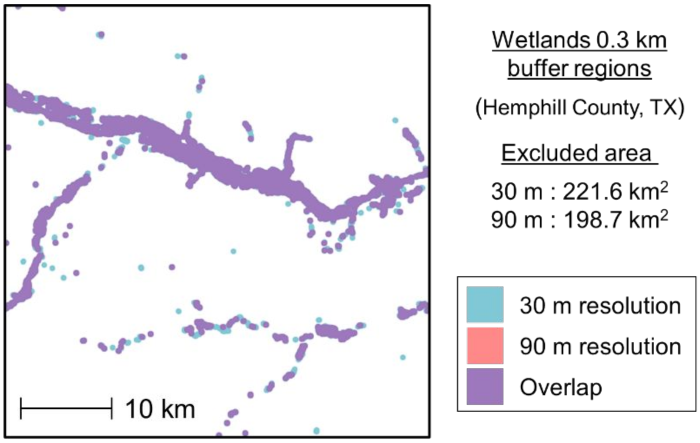

Figure S4. Land excluded by the wetland buffer condition using 30-meter (blue) and 90-meter (red) resolution in Hemphill County, Texas, including overlap between the two regions (purple).

7. Generation technology prioritization

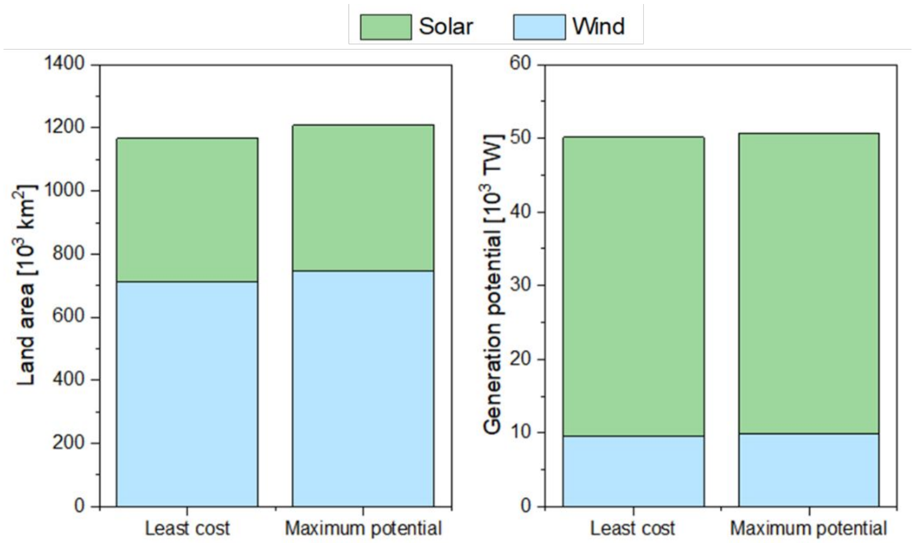

Figure S5. Impact of selecting grid point-level electricity generation technology based on least cost vs. maximum generation potential. Similar results are obtained when comparing land use, generation potential, and median cost of electricity.

8. Cost sensitivity of near- and long-term Ad-DACS

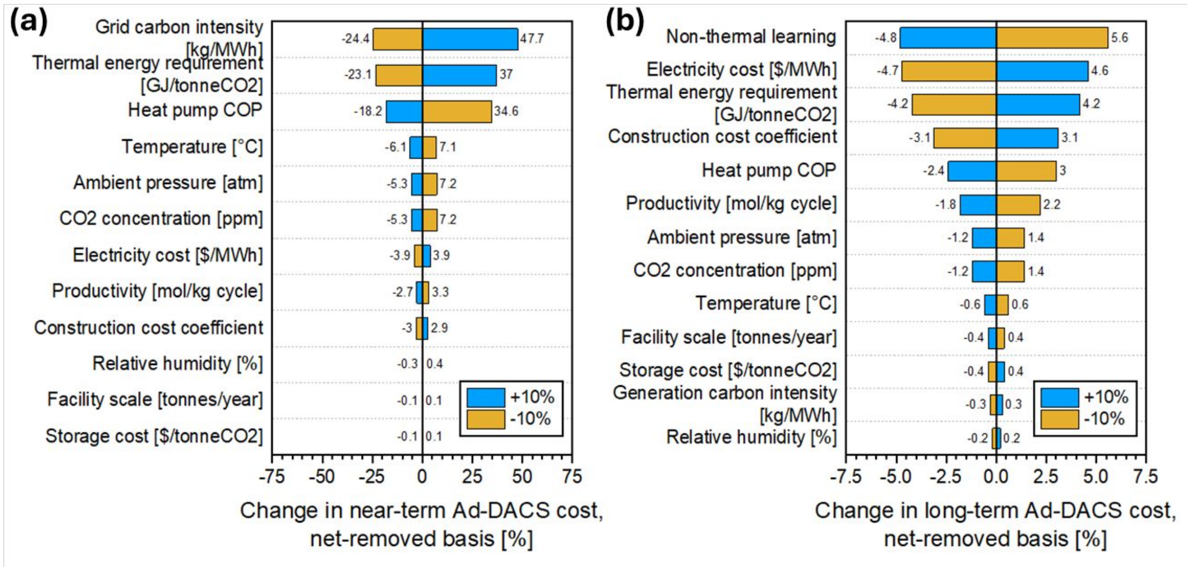

Figure S6. Sensitivity of (a) near-term and (b) long-term Ad-DACS cost to different process parameters, on a net-removed basis. Due to the interactions between temperature and relative humidity on productivity and heat pump efficiency, the latter were also studied independently by manually altering their values, rather than allowing them to be modulated by ambient conditions. "Non-thermal learning" refers to the component learning rates used to estimate long-term costs, without altering learned thermal energy requirements.

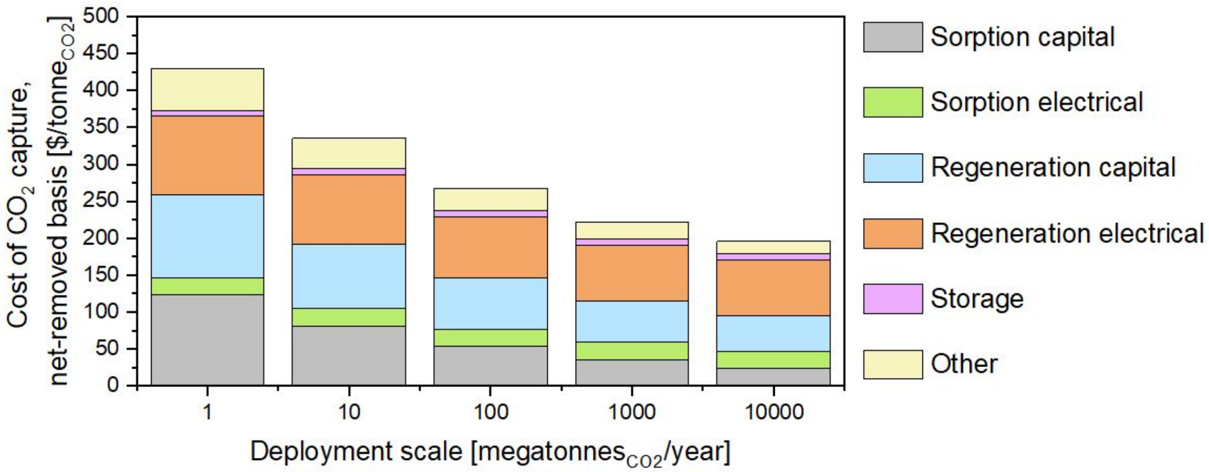

Figure S7: Net-removed cost breakdown of Ad-DACS at different global deployment scales, powered by wind or solar photovoltaic electricity using long-term estimates of electricity costs. All cases assumed plant scales of 100,000 tonnes CO2/year, identical climate conditions, a construction cost coefficient of 1.0, and an energy carbon intensity based on the average between wind and solar photovoltaic.

9. Limitations of this study

The analysis in this work is intended to provide insight into the deployment of adsorbent-based direct air capture and storage in the United States, both in the near- and long-term as we move towards net-zero emissions. To carry out this analysis a number of assumptions were made, and several aspects of DACS deployment were outside the scope of this study and should be addressed in future work.

While the assumptions for siting of energy generation and DACS facilities were relatively restrictive, this work did not account for state- or county-level restrictions and ordinances [11]. The potential overlap in lands utilized for direct air capture and other carbon removal technologies, such as biomass carbon removal and storage (BiCRS) or forest management, was not quantified in this work, but can be found elsewhere [12].

The carbon intensity of energy generation, and by extension CO_2 capture, is quantified in this work; however, the broader environmental impact and burdens of these facilities were not. For example large-scale deployment of direct air capture would require concurrent scale-up of supply chains for raw materials related to the capture media [13], such as reagents for the synthesis of amine-rich polymers [14]. To understand these impacts on cost and viability, full life-cycle assessment (LCA) of the capture facility is required, and will be dependent on the specific technology, the scale of the facility, and its location.

Quantifiable geologic storage was an essential part of this analysis, providing a durable method of sequestering captured CO_2. The utilization of pipelines to transport CO_2 to suitable geologic storage was also not investigated in this work as, due to uncertainties around public perception and their location depending on the location of CO_2 sources and sinks, predicting their long-term deployment is challenging [15]. Other analyses, such as the *Roads to Removal* [16] and *Net Zero America* [17] reports have conducted assessments of CO_2 pipeline infrastructure and its role in achieving net-zero emissions by 2050. There are also analyses of other transport options such as road, rail, and barge, the costs for which vary strongly by the quantity and travel distance of CO_2 being transported [18–20].

10. References

(1) Schlosser, F.; Jesper, M.; Vogelsang, J.; Walmsley, T. G.; Arpagaus, C.; Hesselbach, J. Large-Scale Heat Pumps: Applications, Performance, Economic Feasibility and Industrial Integration. *Renewable and Sustainable Energy Reviews* **2020**, *133*, 110219. https://doi.org/10.1016/j.rser.2020.110219.

(2) Bless, F.; Arpagaus, C.; Bertsch, S. S.; Schiffmann, J. Theoretical Analysis of Steam Generation Methods - Energy, CO 2 Emission, and Cost Analysis. *Energy* **2017**, *129*, 114–121. https://doi.org/10.1016/j.energy.2017.04.088.

(3) U.S. Energy Information Administration (EIA). EIA-861 Annual Electric Power Industry Report: Annual Sales to Ultimate Customers by State and Sector (2010-2022), 2023. https://www.eia.gov/electricity/data/state/ (accessed 2024-08-14).

(4) U.S. Energy Information Administration (EIA). State Electricity Profiles (2023), 2024. https://www.eia.gov/electricity/state/ (accessed 2024-11-25).

(5) U.S. Department of Defense. UFC 3-701-01 DoD Facilities Pricing Guide, with Change 3 | WBDG - Whole Building Design Guide. https://www.wbdg.org/ffc/dod/unified-facilities-criteria-ufc/ufc-3-701-01 (accessed 2023-04-11).

(6) National Renewable Energy Laboratory. *Annual Technology Baseline*. https://atb.nrel.gov/ (accessed 2024-02-20).

(7) NASA Center For Climate Simulation. NASA Earth Exchange Global Daily Downscaled Projections (NEX-GDDP-CMIP6), 2021. https://doi.org/10.7917/OFSG3345.

(8) Cai, X.; Coletti, M. A.; Sholl, D. S.; Allen-Dumas, M. R. Assessing Impacts of Atmospheric Conditions on Efficiency and Siting of Large-Scale Direct Air Capture Facilities. *JACS Au* **2024**, *4* (5), 1883–1891. https://doi.org/10.1021/jacsau.4c00082.

(9) Denholm, P.; Brown, P.; Cole, W.; Mai, T.; Sergi, B.; Brown, M.; Jadun, P.; Ho, J.; Mayernik, J.; McMillan, C.; Sreenath, R. *Examining Supply-Side Options to Achieve 100% Clean Electricity by 2035*; NREL/TP-6A40-81644, 1885591, MainId:82417; 2022; p NREL/TP-6A40-81644, 1885591, MainId:82417. https://doi.org/10.2172/1885591.

(10) Buakhao, W.; Kangrang, A. DEM Resolution Impact on the Estimation of the Physical Characteristics of Watersheds by Using SWAT. *Advances in Civil Engineering* **2016**, *2016*, 1–9. https://doi.org/10.1155/2016/8180158.

(11) Lopez, A.; Cole, W.; Sergi, B.; Levine, A.; Carey, J.; Mangan, C.; Mai, T.; Williams, T.; Pinchuk, P.; Gu, J. Impact of Siting Ordinances on Land Availability for Wind and Solar Development. *Nat Energy* **2023**, *8* (9), 1034–1043. https://doi.org/10.1038/s41560-023-01319-3.

(12) Dai, T.; Ellebracht, N. C.; Hunter-Sellars, E.; Aui, A.; Goldstein, H. M.; Li, W.; Hellwinckel, C. M.; Price, L.; Wong, A. A.; Nico, P.; Basso, B.; Robertson, G. P.; Pett-Ridge, J.; Langholtz, M.; Baker, S. E.; Pang, S. H.; Scown, C. D. Land-Based Resources for Engineered Carbon Dioxide Removal in the United States Exceed the Expected Needs. *One Earth* **2025**, *8* (7), 101349. https://doi.org/10.1016/j.oneear.2025.101349.

(13) Qiu, Y.; Lamers, P.; Daioglou, V.; McQueen, N.; de Boer, H.-S.; Harmsen, M.; Wilcox, J.; Bardow, A.; Suh, S. Environmental Trade-Offs of Direct Air Capture Technologies in Climate Change Mitigation toward 2100. *Nat Commun* **2022**, *13* (1), 3635. https://doi.org/10.1038/s41467-022-31146-1.

(14) McQueen, N.; Gomes, K. V.; McCormick, C.; Blumanthal, K.; Pisciotta, M.; Wilcox, J. A Review of Direct Air Capture (DAC): Scaling up Commercial Technologies and Innovating

for the Future. *Prog. Energy* **2021**, *3* (3), 032001. https://doi.org/10.1088/2516-1083/abf1ce.
(15) Gough, C.; O'Keefe, L.; Mander, S. Public Perceptions of CO2 Transportation in Pipelines. *Energy Policy* **2014**, *70*, 106–114. https://doi.org/10.1016/j.enpol.2014.03.039.
(16) Pett-Ridge, J.; Ammar, H. Z.; Aui, A.; Ashton, M.; Baker, S. E.; Basso, B.; Bradford, M.; Bump, A. P.; Busch, I.; Calzado, E. R.; Chirigotis, J. W.; Clauser, N.; Crotty, S.; Dahl, N.; Dai, T.; Ducey, M.; Dumortier, J.; Ellebracht, N. C.; Egui, R. G.; Fowler, A.; Georgiou, K.; Giannopoulos, D.; Goldstein, H.; Harris, T.; Hayes, D.; Hellwinckel, C.; Ho, A.; Hong, M.; Hovorka, S.; Hunter-Sellars, E.; Kirkendall, W.; Kuebbing, S.; Langholtz, M.; Layer, M.; Lee, I.; Lewis, R.; Li, W.; Liu, W.; Lozano, J. T.; Lunstrum, A.; Mayer, A. C.; Mayfield, K. K.; McNeil, W.; Nico, P.; O'Rourke, A.; Pang, S. H.; Paustian, K.; Peridas, G.; Pilorge, H.; Pisciotta, M.; Price, L.; Psarras, P.; Robertson, G. P.; Sagues, W. J.; Sanchez, D. L.; Scown, C. D.; Schmidt, B. M.; Slessarev, E. W.; Sokol, N.; Stanley, A. J.; Swan, A.; Toureene, C.; Wong, A. A.; Wright, M. M.; Yao, Y.; Zhang, B.; Zhang, Y.; Aines, R. D. *Roads to Removal: Options for Carbon Dioxide Removal in the United States*; LLNL-TR-852901; Lawrence Livermore National Laboratory, 2023. https://doi.org/10.2172/2301853.
(17) Jenkins, J. D.; Mayfield, E. N.; Larson, E. D.; Pacala, S. W.; Greig, C. Mission Net-Zero America: The Nation-Building Path to a Prosperous, Net-Zero Emissions Economy. *Joule* **2021**, *5* (11), 2755–2761. https://doi.org/10.1016/j.joule.2021.10.016.
(18) Oeuvray, P.; Burger, J.; Roussanaly, S.; Mazzotti, M.; Becattini, V. Multi-Criteria Assessment of Inland and Offshore Carbon Dioxide Transport Options. *Journal of Cleaner Production* **2024**, *443*, 140781. https://doi.org/10.1016/j.jclepro.2024.140781.
(19) Stolaroff, J. K.; Pang, S. H.; Li, W.; Kirkendall, W. G.; Goldstein, H. M.; Aines, R. D.; Baker, S. E. Transport Cost for Carbon Removal Projects With Biomass and CO2 Storage. *Front. Energy Res.* **2021**, *9*, 639943. https://doi.org/10.3389/fenrg.2021.639943.
(20) Myers, C.; Li, W.; Markham, G. The Cost of CO2 Transport by Truck and Rail in the United States. *International Journal of Greenhouse Gas Control* **2024**, *134*, 104123. https://doi.org/10.1016/j.ijggc.2024.104123.
